# Indoor Signs Detection for Visually Impaired People: Navigation Assistance Based on a Lightweight Anchor-Free Object Detector

**DOI:** 10.3390/ijerph20065011

**Published:** 2023-03-12

**Authors:** Yahia Said, Mohamed Atri, Marwan Ali Albahar, Ahmed Ben Atitallah, Yazan Ahmad Alsariera

**Affiliations:** 1 Remote Sensing Unit, College of Engineering, Northern Border University, Arar 91431, Saudi Arabia; 2 King Salman Center for Disability Research, Riyadh 11614, Saudi Arabia; 3 Laboratory of Electronics and Microelectronics (LR99ES30), University of Monastir, Monatir 5019, Tunisia; 4 College of Computer Sciences, King Khalid University, Abha 62529, Saudi Arabia; 5 School of Computer Science, Umm Al-Qura University, Mecca 24382, Saudi Arabia; 6 Department of Electrical Engineering, College of Engineering, Jouf University, Sakaka 72388, Saudi Arabia; 7 College of Science, Northern Border University, Arar 91431, Saudi Arabia

**Keywords:** navigation assistance, visually impaired, disabilities, deep learning, object detection, indoor signs

## Abstract

Facilitating the navigation of visually impaired people in indoor environments requires detecting indicating signs and informing them. In this paper, we proposed an indoor sign detection based on a lightweight anchor-free object detection model called FAM-centerNet. The baseline model of this work is the centerNet, which is an anchor-free object detection model with high performance and low computation complexity. A Foreground Attention Module (FAM) was introduced to extract target objects in real scenes with complex backgrounds. This module segments the foreground to extract relevant features of the target object using midground proposal and boxes-induced segmentation. In addition, the foreground module provides scale information to improve the regression performance. Extensive experiments on two datasets prove the efficiency of the proposed model for detecting general objects and custom indoor signs. The Pascal VOC dataset was used to test the performance of the proposed model for detecting general objects, and a custom dataset was used for evaluating the performance in detecting indoor signs. The reported results have proved the efficiency of the proposed FAM in enhancing the performance of the baseline model.

## 1. Introduction

According to the World Health Organization, there are more than 250 million visually impaired persons in the world [1]. Until the present, there were no trusted tools for visually impaired people’s accessibility to indoor environments. Many technologies have been proposed to assist visually impaired people in their indoor navigation. Visually impaired people require more assistance to perform their daily activities compared to other disabilities. People with visual impairment need more effort to learn and interact with their surrounding environments. This motivated a wide range of research to propose a variety of technologies that provide help for visually impaired people. Different feedbacks were provided such as tach via tacti belt, vibration, and audio. Most of those technologies were not standardized, and their distribution is very limited. For example, audio feedback is deployed in elevators and supermarket doors. However, visually impaired people require continuous feedback everywhere for safe and smooth navigation in indoor spaces.

Recently, autonomous systems were widely deployed in many fields such as automotive [2,3], aerial navigation [4,5], and healthcare entertainment. This was due to the success of new computer vision techniques. As image processing is the most dominating task, most autonomous systems rely on it to achieve the required performance. In effect, there was intensive innovation in this research field. Deep learning techniques were the most powerful method for image processing until today, which are based on an artificial neural network with deep structure. In particular, convolutional neural networks (CNNs) are the most dominating for image processing applications. CNNs are inspired by the biological brain and have the ability to learn directly from input data without the need for handcrafted features. Existing works proved the efficiency of the CNN models for image classification [6], scene recognition [7], object detection [8], traffic sign recognition [9], indoor object detection [10], pedestrian detection [11], traffic light detection [12], and many others. However, the research on the assistance of visually impaired people is limited and few methods have been proposed.

Indoor navigation presents a serious challenge for visually impaired people. The design of image processing applications for assisting the navigation of visually impaired people is still far away due to different challenges. External challenges such as complex backgrounds, illumination variation, occlusion, and deformation are the most to handle. Internal challenges such as the limit of computation resources and the limit of power must be solved to achieve the needed performances. The proposed application must provide accurate and real-time feedback while being suitable for implementation on low-power devices such as smartphones or dedicated platforms.

The main goal of this work is to design an indoor sign detection application to be used for the assistance of visually impaired people’s navigation. Building a powerful indoor sign detection application requires great effort and a precise balance between computation complexity and performance. Most of the existing object detection models were improved to achieve high detection precision and fast inference time that meet real-time constraints without considering computation complexity. Object detection methods can be categorized into two main categories. One-stage methods such as you look only once (YOLO) [13] and single shot multi-box detection (SSD) [14] perform the classification repression tasks using a single CNN model. They were designed for fast processing but achieve medium precision. Two-stage methods such as faster RCNN [15] and mask RCNN [16] use a regional proposal network (RPN) to generate regions of interest alongside the classification model. This category achieves higher precision compared to one-stage methods but presents a slow processing speed. The main common point between the two categories is that they are based on anchors for the localization task. Subsequently, the performance of the detection model is heavily related to the optimization of anchor hyperparameters such as scale, size, and aspect ratio. As the optimization of those hyperparameters requires intensive manual calibration, it is easy to achieve high performance.

To avoid the process of anchor hyperparameters calibration, anchor-free object detection methods [17] were proposed as a flexible alternative to anchor-based one-stage methods. Anchor-free methods eliminate the need for predefined anchors, which makes them adaptable to a wide diversity of target objects. For indoor sign detection, anchor-free methods achieved low precision due to the small size of the detected signs and the complexity of the background. The achieved precision can be improved by removing the interference caused by background features. As mentioned earlier, two-stage object detection methods achieved higher detection precision due to the use of the RPN that separates the foreground from the background. RPN removes the interference of complex background features and helps the model to focus only on relevant features of the foreground objects. Inspired by the RPN, we proposed a foreground attention module (FAM) to be used with the anchor-free object detection model centerNet [18]. Furthermore, we proposed the use of a lightweight backbone to achieve lower computation complexity. The main idea was to achieve high detection precision with low computation complexity.

This work proposed FAM-CenterNet, which used foreground information to reduce the influence of complicated background features in indoor navigation scenarios. The semantic segmentation that underpins the foreground region proposal network is supervised with the segmentation label obtained from the bounding box label. The transition between the foreground and background is introduced as the midground, which can give extensive edge information about the objects. For the incorporation of both the foreground position and scale information, detection accuracy is effectively improved. To balance the performance of the model and its computation complexity, the DLA [19] backbone was introduced which already achieved high recognition accuracy with a very low computation complexity. The combination of the proposed FAM-CenterNet and DLA was very effective, and the experimental results will prove this assumption.

In summary, the main contribution of this work is using the centerNet model with a light backbone to guarantee low computation complexity. The proposed backbone was modified to achieve high detection accuracy. The original DLA model has a low computation complexity and acceptable accuracy. To improve the accuracy, we proposed to replace regular convolution with deformable convolution. This modification resulted in increased accuracy with a slight increase in computation complexity. Furthermore, an attention module was used to improve the localization performance of a small object in a complex background. The proposed attention module was inspired by the RPN in two-stage detectors. The RPN was very effective in detecting an object under challenging conditions such as a complex background, small object, and occlusion. The proposed FAM was designed to perform the same function as the RPN with lower computations.

The proposed approach has been evaluated on two datasets. First, the Pascal VOC dataset [20] was used to evaluate the proposed FAM-centerNet for general object detection to prove its performance. Second, the model was evaluated on the dataset proposed in [21] for indoor sign recognition as the main goal of this is to build an indoor sign detection system for assisting visually impaired people in their navigation. The evaluation of the proposed model proved its efficiency with a detection mAP of 94.52%. Compared to the state-of-the-art model, the proposed model outperforms the SSD model by more than 14% and the YOLO v3 model by more than 12%. Furthermore, the proposed model presents an improvement of more than 3% compared to the centerNet model.

The main contributions of this work are:

- Building an indoor sign recognition system for the navigation assistance of visually impaired people.
- Proposing a foreground attention module to enhance the performance of an anchor-free object detection model.
- Introducing the use of midground for transition between the background and foreground to collect relevant edge features of the target object.
- Introducing the use of foreground scale features for the regression to enhance the performance of the localization process.
- Proposing the use of a lightweight backbone to balance the performance and the computation complexity.
- Evaluating the proposed FAM-centerNet on two datasets. Extensive experimentations confirmed the efficiency of the proposed model and achieved top performances in both precision and inference speed while obtaining comparable computation complexity.

The remainder of this paper is arranged as follows: Section 2 presents related works. In Section 3, the proposed approach is described and detailed. Experiments and results are presented and discussed in Section 4. In Section 5, conclusions and future works are provided.

## 2. Related Works

For a long time, object detection has been an active research field that attracts industrial and academic research. Thus, important achievements were reported, and a wide range of methods was proposed. Classical machine learning methods were based on handcrafted features techniques, which are time-consuming with low performance. Recent methods based on deep learning techniques have achieved great success, especially using CNN models. There are two main categories in object detection models, which are: anchor-based models and anchor-free models.

Anchor-based models rely on anchor information for both classification and regression. Those models can be classified into two sub-categories which are one-stage detectors and two-stages detectors. Two-stage detectors such as faster RCNN [15] and mask RCNN [16] are based on a classification network and region proposal network (RPN) that work jointly. The main role of the RPN is to generate regions of interest that may contain target objects to reduce the processing time by eliminating background processing and focusing on foreground objects. The use of the RPN was very effective in achieving high detection accuracy but present a slow processing time that does not respect real-time constraints. One-stage detectors solve the problem by eliminating the RPN to accelerate the processing time. Models such as Yolo [13] and SSD [14] have achieved real-time processing with acceptable precision. The main problems of anchor-based models are that they require a huge effort to be fine-tuned, have difficulties setting predefined anchors for specific target objects, and are computationally extensive.

Anchor-based models eliminated the need for predefined anchors with the estimation of keypoints for the detection [17,18,22]. Those models can be classified into two subcategories which are center point estimation and multiple points estimation. Models with multiple keypoints estimation use many points to locate the target object. cornerNet [17] uses two corner points, the bottom left corner and the top right corner, to predict bounding boxes. This estimation is very sensitive to edges and generates many false predictions. To achieve high precision, keypoint triplets were proposed in [18] by introducing more geometric constraints. This model is based on the early cornerNet and estimates additional keypoint at the center of the predicted bounding box and then verifies if the center point coincides with the center area defined by the corner points. Another solution was proposed by extremeNet [23], which estimates the four corner points of the bounding box in addition to the center point. This estimation results in achieving more detection precision. RepPoints [24] proposes the estimation of nine keypoints to enhance the precision. A weak supervision technique was used to estimate the keypoints. Increasing the number of the estimated keypoints can effectively enhance the detection precision. However, a combinatorial grouping stage is required after the keypoints estimation, which results in a slow inference speed.

Models with center point estimation only estimate the center point for each target object and use it directly for the final prediction without any grouping stage. Densebox [25] is based on a fully convolutional neural network that estimates the probability of a pixel as a center point and guesses the relative location of the two corners of the predicted bounding box centered on it. A center point estimation with scale prediction was proposed in [26]. Semantic features [27] were used to generate a center point heatmap and prediction map for object detection. FCOS [22] has replaced the anchors in RetinaNet [28] with center point estimation. A features pyramid network (FPN) was used for detection at different scales. In addition, centerness estimation was added to guide the training.

There is a particular object detection model that takes advantage of semantic features. Mask RCNN [16] is an object detection model that generates output bounding boxes and semantic segmentation simultaneously. It was proved that multi-task training enhances detection performance. Object detection models were trained using semantic segmentation annotations in [16,29] and good results were achieved. In [30], semantic features and detection features were concatenated at a high level to improve the prediction. In addition, DES [31] replaces the concatenation with activation for features combination at a low level. All those works are built on anchor-base models. For example, the work in [30] is based on faster RCNN and the DES [31] used the SSD as the baseline.

An indoor object detection system was proposed in [32]. The proposed system is based on Yolo v3 [33]. The proposed system was developed to detect indoor objects such as doors, stairs, tables, and so on. Objects that affect the safety of blind and visually impaired people in the indoor environment were considered. A custom dataset was collected and labeled for training and evaluating the YOLO v3 model. According to the reported results, good detection accuracy was achieved, but the model was computationally extensive and required a high-performance GPU to run smoothly.

Indoor sign detection was not widely investigated. Afif et al. [21] proposed an indoor sign detection for wayfinding and navigation assistance of visually impaired people based on the RetinaNet model [28]. This model was used to solve the problem of unbalanced training data. To evaluate the proposed model, a custom dataset was collected and manually annotated. A mean average precision (mAP) of 93.45% was achieved with an inference speed of 25 FPS. The proposed approach achieved high performance, but it was computationally extensive and implemented on a high-performance GPU, which makes it non-suitable for real-world applications.

After a deep analysis of the literature, it is obvious that anchor-free models are more suitable for implementation on embedded devices due to their high performance and low computation complexity. On the other hand, two-stage models are more accurate in detection and more trusted. Furthermore, exploring semantic features can improve the performance without affecting the computation complexity. In this work, we proposed the use of an anchor-free object detection model enhanced with a foreground attention module inspired by the RPN of two-stage models. In addition, semantic features were explored to extract foreground features, and their location was used to enhance the regression output. A detailed description of the proposed model is presented in the next section.

## 3. Materials and Methods

**Figure 1:**
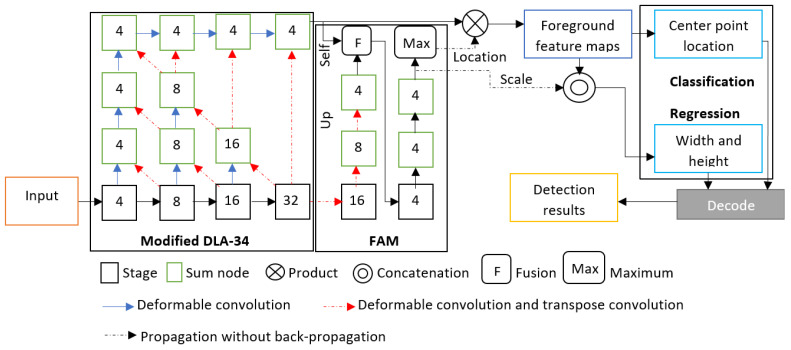
The proposed FAM-centerNet. The numbers inside the boxes refer to the stride of the image. Modified DLA-34 was introduced using deformable convolution to change channels and transpose convolution for upsampling.

The proposed FAM-centerNet is based on an improved anchor-free object detection model. CenterNet was the baseline, and an additional foreground attention module (FAM) was introduced to collect the foreground features. As a backbone, an improved deep layer aggregation (DLA) model [19] was used to guarantee high performance and low computation. The DLA-34 has the lowest computation overhead in the DLA family, which makes it the most suitable for implementation in embedded devices. The DLA model is designed for image classification with a novel hierarchical skip connection scheme. To gain more performance and to make the model more adaptable to the target objects, regular convolution layers at every upsampling stage were replaced with deformable convolution layers [34] with a 3 × 3 kernel. Figure 1 illustrates the proposed FAM-centerNet.

The integration of the FAM presented a serious problem. The use of the DLA structure has facilitated the integration of the self- and up-branches of the FAM. In addition, the up-branch was designed using deformable convolution and transpose convolution instead of regular convolution. The self-branch was directly injected into the fusion operation from the top of the DLA structure.

The FAM was inspired by the RPN [15] to estimate foreground regions. By collecting foreground location features, feature maps for the foreground can be generated using the output of the DLA-34 used for feature extraction. The foreground feature map (FFM) can be computed using an element-wise multiplication between the feature maps (FMs) of the DLA-34 and the proposal foreground region (F).

Considering an input image with width *W* and height *H*, the produced center point heatmap in the classification is $P\in {\left[0,1\right]}^{\frac{W}{R}\times \frac{H}{R}\times C}$, where *R* is the output stride and *C* is the class of the detected object (indoor sign in this work). So, a detected center point corresponds to a prediction $P_{xyc}=1$. To generate the ground truth center point heatmap $G_{p}\;\in {\left[0,\;1\right]}^{\frac{W}{R}\times \frac{H}{R}\times C}$, a Gaussian kernel is computed as Equation (1):

(1) $$G_{P_{xyc}}=\exp(-\frac{{\left(x-\hat{cp_{x}}\right)}^{2}+{\left(y-\hat{cp_{y}}\right)}^{2}}{2\;\sigma_{\hat{cp}}^{2}}$$

where $\hat{cp}=\frac{cp}{R}$ is the equivalent low-resolution point of the ground truth center point cp∈R of category *C* and $\sigma_{\hat{cp}}$ is a size-adaptive deviation in the detected object [17]. In the inference, the center point is estimated using the top 100 peaks with a value less than its 8 connected neighbors in the generated heatmap.

For the regression task, a scale prediction map $S\in {\left[0,1\right]}^{\frac{W}{R}\times \frac{H}{R}\times 2}$ is generated. The scale prediction map for a predicted center point $p_{k}$ is $S_{k}=\left(w_{k},\;h_{k}\right)$, where $w_{k}$ and $h_{k}$ are the width and height of the object, respectively, with center $p_{k}$. In addition, the centerNet model predicts the local offset $O\in {\left[0,1\right]}^{\frac{W}{R}\times \frac{H}{R}\times 2}$. A shared offset was affected to all classes to recover the discretization error resulting from the output stride. For an estimated center point $p_{k}$, the model predicts an offset $O_{k}=\left(\delta_{k}{}_{x},\;\delta_{k}{}_{y}\right)$. To generate the final prediction of the bounding box, the coordinate of its corners is determined using the center point $p_{k}$ and scale map $S_{k}$. The coordinate of the bounding box can be computed as Equation (2).

(2) $$\begin{array}{l} x_{k_{1}}=p_{k_{x}}+\delta_{k_{x}}-\frac{w_{k}}{2}\\ y_{k_{1}}=p_{k_{y}}+\delta_{k_{y}}-\frac{h_{k}}{2}\\ x_{k_{2}}=p_{k_{x}}+\delta_{k_{x}}+\frac{w_{k}}{2}\\ y_{k_{2}}=p_{k_{y}}+\delta_{k_{y}}+\frac{h_{k}}{2} \end{array}$$

After presenting the main concept of the proposed FAM-centerNet model, we will move to present the proposed foreground attention module (FAM) in Section 3.1. Section 3.2 describes how to take advantage of the scale information for the regression task. The concept of midground use is presented in Section 3.3. In Section 3.4, we present the training process and the design of the loss function. Section 3.5 provides an overview of the method of generating foreground segmentation labels.

### 3.1. Foreground Attention Module

The proposed FAM is based on semantic segmentation with the fusion of two methods. The first is based on the up-branch and the second is based on the self-branch. As shown in Figure 1, the up-branch is based on an upsample process of low resolution, and the self-branch is connected to high-resolution features. Since both methods are useful and selecting the best one is not feasible theoretically, we proposed to fuse the outputs of both methods for further processing. After the fusion process, a set of three convolution layers were applied followed by a pixel-wise maximum operation to generate the final foreground feature maps.

#### 3.1.1. FAM Using Up-Branch

For the up-branch, the feature maps of the decoder were upsampled based on consecutive convolution layers. The resulting feature maps were used to obtain the foreground features maps. In effect, deformable convolution layers were used to change the channels, and the transpose convolution layers were used to upsample the feature maps. This method of the encoder–decoder strategy was widely used for semantic segmentation tasks, and it was adopted in this work to take advantage of the semantic features. Assuming that *E* is the output feature maps of the encoder and *D* is the convolution layers applied to those feature maps, the output *F* of the up-branch method can be computed as Equation (3).

(3) F=FAM(D(E))

#### 3.1.2. FAM Using Self-Branch

The self-branch directly applied regular convolution layers to obtain the foreground feature maps. In this method, the feature maps were collected from the top of the modified DLA-34 and then further processing was performed. Considering that *FM* is the output feature map of the top of the DLA-34, the foreground feature map *F* can be computed as Equation (4).

(4) F=FAM(FM)

### 3.2. Scale Information

Since the proposed FAM is based on object categories, the scale information for each object can be provided for each category. Using scale information for the regression part will improve the performance. So, region proposals from the foreground feature maps were concatenated with the scale information to be fed to the regression process. This technique will enhance the regression and adapt the output bounding boxes to the target objects.

### 3.3. Midground Integration

**Figure 2:**
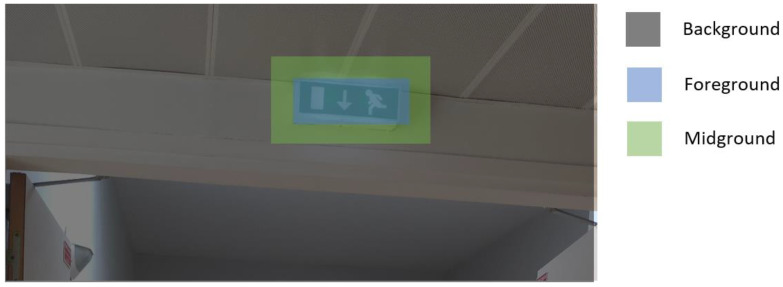
Presentation of background, foreground, and midground in the image.

For two-stage detectors, each proposal region is classified as foreground or background. In RPN-based models, a region with an overlap of less than 0.3 is a background, a region with an overlap of more than 0.7 is a foreground, and the region between them is ignored. The ignored regions are considered midground in this work. To perfectly localize an object, the generated bounding boxes are too close to the edges of the object. So, the generated segmentation labels are strict foreground regions, and the surrounding area of the object was not considered. Since the surrounding area contains edge information, collecting those features is very important for both regression and classification. The midground region is the surrounding area of the foreground area. Figure 2 presents the difference between background, midground, and foreground.

### 3.4. Semantic Label Generation

**Figure 3:**
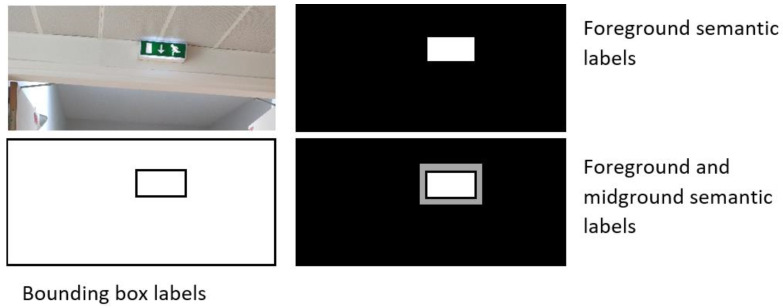
Semantic label generation.

The semantic labels were generated to be used for training the proposed FAM. Following the strategy in [35], the annotations are segmentation boxes. To generate the annotations, ground-truth bounding boxes were projected on the location of the object under the output stride. If the projected bounding box locates an object, the pixels in annotation are set to one, otherwise they will be set to zero. The size of the segmentation box is $\frac{H}{R}\times \frac{W}{R}\times C$ where *C* is the categories of the target dataset, *R* is the output stride, and H and W are the height and width of the input image, respectively. An illustration of the semantic label generation is presented in Figure 3.

### 3.5. Training Strategy

**Figure 4:**
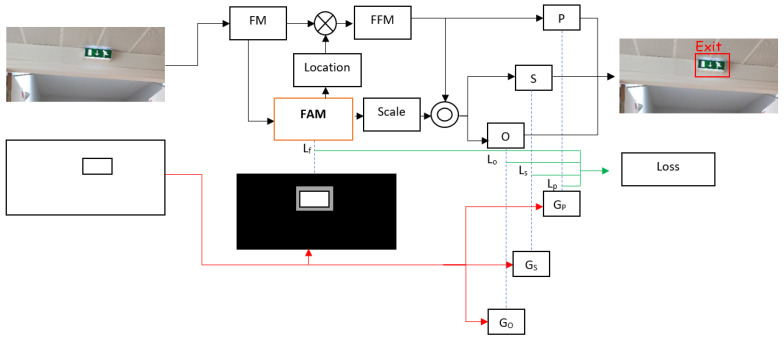
Training workflow of the proposed FAM-centerNet.

The training of the proposed FAM-centerNet is based on bounding box labels and the generated semantic labels of foreground and midground. The training workflow is presented in Figure 4.

To train the model, a custom loss function was proposed. The loss function is composed of four loss parts. A loss for the proposed FAM noted *L_f_*, a loss for the center point prediction noted *L_p_*, a loss for the scale prediction *L_S_*, and a loss for the offset prediction. The loss of the proposed FAM can be computed as Equation (5):

(5) $$L_{f}=\frac{-1}{N}\sum_{xyc}\{\begin{array}{l} {\left(1-F_{xyc}\right)}^{\alpha }\log\left(F_{xyc}\right)\;if\;G_{f_{xyc}}=1\\ {\left(1-G_{f_{xyc}}\right)}^{\beta }{\left(F_{xyc}\right)}^{\alpha }\log\left(1-F_{xyc}\right)\;otherwise \end{array}$$

where $F_{xyc}$ is a foreground proposal, $G_{f_{xyc}}$ is the associated ground truth for this proposal, N is used for normalization, which is the number of pixels for $G_{f_{xyc}}=1$, and α and β are hyperparameters to be tuned.

The loss for the center point prediction can be computed as Equation (6):

(6) $$L_{P}=\frac{-1}{N}\sum_{xyc}\{\begin{array}{l} {\left(1-P_{xyc}\right)}^{\alpha }\log\left(P_{xyc}\right)\;if\;G_{P_{xyc}}=1\\ {\left(1-G_{P_{xyc}}\right)}^{\beta }{\left(P_{xyc}\right)}^{\alpha }\log\left(1-P_{xyc}\right)\;otherwise \end{array}$$

where $G_{P_{xyc}}$ is the ground truth for a predicted center point and $P_{xyc}$ is a predicted center point.

The loss for the scale prediction can be computed as Equation (7):

(7) $$L_{s}=\frac{-1}{N}\sum_{k=1}^{N}\left|S_{k}-G_{s_{k}}\right|$$

where $S_{k}$ is the predicted scale for a center point $p_{k}$ and $G_{s_{k}}$ is the corresponding ground truth.

The loss for the offset prediction can be computed as Equation (8):

(8) $$L_{o}=\frac{-1}{N}\sum_{k=1}^{N}\left|O_{k}-G_{o_{k}}\right|$$

where $O_{k}$ is the predicted offset for a center point $p_{k}$ and $G_{s_{k}}=\frac{cp}{R}-\hat{cp}$ is the corresponding ground truth.

In summary, the total loss *L* is the weighted sum of all losses. The total loss can be computed as Equation (9):

(9) $$L=\lambda_{f}L_{f}+\lambda_{p}L_{p}+\lambda_{s}L_{s}+\lambda_{o}L_{o}$$

where λ is the weight associated with each loss.

## 4. Results

The evaluation of the proposed FAM-CenterNet was performed on two datasets, the Pascal VOC dataset for general object detection and a custom dataset for indoor sign detection. All experiments were conducted on a desktop equipped with an Intel i7 CPU (Intel Core, Santa Clara, CA, USA), 32 GB of RAM, and Nvidia GTX 960 GPU. The model was developed based on a Pytorch deep learning framework with GPU support based on CUDA 10 (NVIDIA, Santa Clara, CA, USA), and cuDNN v7 (NVIDIA), acceleration.

### 4.1. Implementation Details

First, we started with evaluating the proposed model using the Pascal VOC dataset. The Pascal VOC 2012 dataset contains a total of 11,530 images with 27,450 annotated objects for training and validation and 5585 images were used for testing. The Pascal VOC 2012 does not provide annotations for the testing data. The main objective of this dataset is the detection of a set of objects, 20 categories considered, in a real scene. A wide variety of annotated objects were provided with different sizes, distances, orientations, illumination, and occlusion. For training and evaluation of the proposed model, the original resolution of images was resized to 384 × 384. The Adam optimizer was used as a learning algorithm with an initial learning rate of 10^−4^ with a drop at the 90th epoch to 10^−5^ and the 120th epoch to 10^−6^. A batch size of 8 was used and the model was trained for 150 epochs. Many data augmentation techniques were applied such as flipping, rotation, and translation.

Second, an indoor sign detection dataset was used to evaluate the model for our target task, which is detecting indoor signs for navigation assistance for visually impaired people. This dataset was collected for different conditions by considering many challenges such as occlusion, the difference in point of view, lighting conditions, and orientation. A total of 4000 images were collected and divided into training and testing sets where 2600 images were used for training and 1400 were used for testing. The dataset provides annotations for four classes which are WC, exit, disabled exit, and confidence zone. Those signs are the most important for regular navigation in indoor environments. The input images were resized to 416 × 416 for both training and testing. To train the proposed model, the Adam optimizer was used with an initial learning rate of 1.5 × 10^−4^ with a drop at the 70th epoch to 1.5 × 10^−5^ and a drop at the 100th epoch to 1.5 × 10^−6^. The model was trained for a total of 120 epochs with a batch size of 8. The model was initialized with the pre-trained weights on the Pascal VOC dataset. For the evaluation using both datasets, the mean average precision (mAP) was used as an evaluation metric.

### 4.2. Evaluation

The evaluation of the proposed model was performed at different configurations of the FAM. As explained earlier, two methods for feature collection with two fusion techniques were proposed for the FAM. The first method is based on the up-branch by upsampling the features and the second method is based on the self-branch that directly collects features from the backbone. For fusion, we proposed summation and concatenation to merge features.

The baseline for this work is the centerNet model, and the proposed FAM with different configurations was applied separately to evaluate the influence of each one. The achieved results using the Pascal VOC 2012 are presented in Table 1. By looking at the achieved results, it can be seen that all methods proposed for the FAM with all fusion techniques outperform the baseline model by at least 2% in the mAP. The reported results proved the effectiveness of the proposed FAM. Based on the results presented in Table 1, the fusion with the concatenation technique achieved the best results, and the self-branch method is better than the up-branch method. The fusion with the concatenation technique has been improved by 4.63% compared to the baseline model. Furthermore, the detection rate of small objects has been widely improved due to the elimination of complex background interference using the FAM. The fusion techniques also improved the performance. The concatenation technique maintains the entire features collected from the backbone, while the summation technique sums up the collected features which cause a loss in information. The summation technique is a particular case of the concatenation technique for convolution with multi-channels, where the convolution weights are the same for all channels and the connection weights are set to one.

After that, we moved to evaluate the proposed model with different FAM configurations using the indoor sign detection dataset. Similar to the Pascal VOC dataset, we evaluate each configuration separately to observe its influence on the detection rate. The achieved results are presented in Table 2.

As shown in Table 2, all configurations of the proposed FAM outperform the baseline model, which proves the effectiveness of our contribution. Then, by evaluating the two proposed methods and the fusion techniques, it was discovered that the self-branch method achieved the best results for exit and WC signs with an improvement of 1.7% and 2.1% compared to the baseline model, respectively. Thus, it was proved that the proposed FAM eliminated the interference from complex backgrounds.

The fusion with the concatenation technique has the most balanced performance with an average improvement of 2% compared to the baseline. Different fusion techniques have achieved variable performances in different categories. The proposed fusion with the concatenation technique saves information generated by earlier layers, while the fusion with the summation technique accumulates the output of previous layers, which leads to losing some relevant features and decreases performance. Therefore, the fusion with the concatenation technique is more powerful and useful for the detection of small objects in complex background scenes. Hence, this configuration has achieved the best performance.

While the proposed enhancement improves the detection rate, the inference speed decreases. Table 3 presents the inference speed for each configuration. It can be seen that the self-branch has the fastest inference speed and the fusion with concatenation has the lowest speed.

After a deep analysis of the achieved results, the configuration with the self-branch was identified as the most suitable for indoor sign detection for navigation assistance for visually impaired people. The main reasons for this choice are the following:

The FAM with self-branch configuration has the lowest computation complexity. In effect, it does not require any additional layers or operations since it is directly connected to the output feature maps from the backbone. Correspondingly, it will be suitable for implementation on embedded devices and can meet the real-time constraints of the target application.

The detection rate of the model is comparable to other configurations. The generated information of the FAM is based directly on the feature maps from the backbone, which may lead to paying more attention to foreground regions. This enhances the detection performances for both classification and localization.

Experimental results have proved the effectiveness of the proposed FAM for enhancing the performance of an anchor object detection model by eliminating the interference of a complex background and making the model pay more attention to the foreground regions. Considering that the exit and WC signs are most frequent and useful for navigation assistance of visually impaired people compared to other signs, and considering the importance of real-time processing, the self-branch method is the best configuration for this application.

### 4.3. Ablation Study

In this section, we will investigate the impact of the scale information and midground. Considering the proposed FAM-centerNet as a baseline model, the introduction of scale information and midground was evaluated on the indoor sign detection dataset. The achieved results of the baseline and the proposed improvement were reported in Table 4.

The reported results proved the efficiency of the scale information introduction for improving performance. The improvements for the exit sign are more obvious. The detection rate for the exit was improved by 1.49%. In addition, the detection rate for other signs has been slightly improved. The impact of the midground introduction was demonstrated by achieving slight improvements compared to the FAM-centerNet. An improvement in mAP by 0.36% was achieved. For the disabled exit sign, no improvements were achieved. The combination of scale information and midground was very effective, and great results were achieved. The model achieved the top detection rate of 94.52% mAP. The proposed improvements were very effective, but the midground introduction presents a lower impact on the performance.

## 5. Comparison and Discussion

To prove the achieved results, a comparison study against state-of-the-art models was performed. Considering the difference in the experimental environment, the existing model was reproduced in our experimental environment for a fair comparison. Table 5 presents a comparison against the state-of-the-art models on indoor sign detection.

As shown in Table 5, the proposed FAM-centerNet outperforms the state-of-the-art models for all signs. The proposed model outperforms the RetinaNet [21] by 1.7% in terms of mAP. The detection rate of the WC sign was the most improved by 1.39% compared to the other signs. In terms of speed, the FAM-ceneterNet model achieved a slightly lower speed of 85 ms compared to 80 ms for the RetinaNet model [21]. Considering the achieved improvement in mAP, the proposed model is very effective for detecting indoor signs for integration into navigation assistance systems for visually impaired people.

Finally, the performance of the proposed model was compared to state of the-art object detection models including SSD, centerNet, RetinaNet and YOLO v3. The comparison was performed using the same dataset and experiment environment. The DLA 34 backbone was considered for all object detection models for a fair comparison. Table 6 presents a comparison between the proposed model and state-of-the-art object detection models. The reported results show that the proposed model has the best detection accuracy with a good processing speed and a comparable computation complexity. The number of parameters in the proposed model is slightly greater than YOLO v3 while achieving more than 12% higher mAP. Based on the aforementioned, the proposed model achieved a good trade-off between accuracy and speed while being computationally efficient.

## 6. Conclusions

Navigation assistance for visually impaired people in indoor spaces requires high performances detection system. Considering the enhancement of an anchor-free model for indoor sign detection, a FAM-centerNet model was proposed. It was based on the centerNet model with a foreground attention module to eliminate the interference of complex backgrounds in indoor images. The FAM takes advantage of semantic segmentation features generated based on the bounding box labels. In addition, the midground was introduced to make the transition between the foreground and background. The proposed improvements have enhanced the detection rate compared to the centerNet model. Experimental results using the Pascal VOC dataset and indoor sign detection dataset demonstrated the high performance of the proposed model. In future work, the model can be compressed to be implemented on embedded devices and tested under realistic conditions.

## Figures and Tables

**Table 1:** Achieved results using the Pascal VOC 2012 dataset.

Class	AP (%)				
	Baseline	Up-Branch	Self-Branch	Fusion: Summation	Fusion: Concatenation
aeroplan	86.28	88.34	89.27	90.39	91.36
bicycle	89.20	91.12	92.41	93.34	94.11
bird	84.78	86.77	87.56	88.81	89.79
boat	75.55	77.42	78.45	79.51	80.48
bottle	72.70	74.19	75.38	76.46	77.93
bus	92.67	95.89	96.82	97.33	97.89
car	87.22	89.37	90.28	91.32	92.23
cat	86.27	88.39	89.48	90.37	91.44
chair	69.50	71.67	72.61	73.53	74.49
cow	90.78	92.86	93.67	94.72	95.12
dining table	81.19	83.41	84.44	85.39	86.21
dog	84.71	86.73	87.46	88.37	89.91
horse	85.47	87.32	88.47	89.56	90.28
motorbike	87.40	89.46	90.18	91.48	91.43
person	88.77	90.63	91.29	92.84	93.29
potted plant	57.41	59.78	60.89	61.47	62.48
sheep	86.48	88.75	89.92	90.89	91.38
sofa	71.88	73.77	74.93	75.28	76.33
train	79.41	81.56	82.51	83.36	84.19
tv monitor	75.10	77.59	78.69	79.26	80.25
**mAP**	81.63	83.75	84.76	85.69	86.26

**Table 2:** Achieved detection rate using the indoor sign detection dataset.

Class	AP (%)				
	Baseline	Up-Branch	Self-Branch	Fusion: Summation	Fusion: Concatenation
exit	92.97	93.72	94.67	94.36	94.56
WC	92.83	92.95	94.93	94.34	94.87
disabled exit	92.91	93.12	93.34	93.81	94.69
confidence zone	91.36	91.68	91.89	92.95	93.97
**mAP**	92.51	92.86	93.7	93.86	94.52

**Table 3:** Achieved inference speeds.

FAM Configurations	Speed (ms)
Baseline	69
Up-branch	81
Self-branch	80
Fusion with summation	82
Fusion with concatenation	85

**Table 4:** Ablation study on the impact of the scale information and midground use.

Class	AP (%)			
	FAM-centerNet	With Scale Information	With Midground	With Scale Information and Midground
Exit	92.36	93.58	93.46	94.56
WC	92.82	92.93	92.91	94.87
Disabled exit	92.88	93.34	92.88	94.69
Confidence zone	91.92	91.98	92.15	93.97
**mAP**	92.49	92.95	92.85	94.52

**Table 5:** Comparison against the state-of-the-art model.

Class	mAP (%)	
	RetinaNet [21]	FAM-centerNet
Exit	93.52	94.56
WC	93.48	94.87
disabled exit	93.83	94.69
confidence zone	92.97	93.97
**Map**	93.45	94.52

**Table 6:** Comparison against state-of-the-art object detection models.

Model	mAP (%)	Speed (ms)	FLOPs (G)	Parameters (M)
SSD	80.12	63	34.65	1.3
YOLO v3	81.58	57	38.21	1.7
RetinaNet	90.03	80	67.66	2.4
CenterNet	91.26	69	52.48	1.8
FAM-centerNet (ours)	94.52	85	55.27	1.9

## Data Availability

Data will be made available on request.
